# Leukocyte-Associated Immunoglobulin-like Receptor-1 is regulated in human myocardial infarction but its absence does not affect infarct size in mice

**DOI:** 10.1038/s41598-017-13678-5

**Published:** 2017-12-21

**Authors:** Guilielmus H. J. M. Ellenbroek, Judith J. de Haan, Bas R. van Klarenbosch, Maike A. D. Brans, Sander M. van de Weg, Mirjam B. Smeets, Sanne de Jong, Fatih Arslan, Leo Timmers, Marie-José T. H. Goumans, Imo E. Hoefer, Pieter A. Doevendans, Gerard Pasterkamp, Linde Meyaard, Saskia C. A. de Jager

**Affiliations:** 1 Laboratory of Experimental Cardiology, University Medical Center Utrecht, Utrecht, The Netherlands; 2 Department of Medical Physiology, University Medical Center Utrecht, Utrecht, The Netherlands; 3 Department of Cardiology, University Medical Center Utrecht, Utrecht, The Netherlands; 4 Department of Clinical Chemistry and Haematology, University Medical Center Utrecht, Utrecht, The Netherlands; 5 Netherlands Heart Institute, Utrecht, The Netherlands; 6 Department of Molecular Cell Biology, Leiden University Medical Center, Leiden, The Netherlands; 7 Laboratory of Translational Immunology, Department of Immunology, University Medical Center Utrecht, Utrecht, The Netherlands

## Abstract

Heart failure after myocardial infarction (MI) depends on infarct size and adverse left ventricular (LV) remodelling, both influenced by the inflammatory response. Leukocyte-associated immunoglobulin-like receptor 1 (LAIR-1) is an inhibitory receptor of ITAM-dependent cell activation, present on almost all immune cells. We investigated regulation of LAIR-1 leukocyte expression after MI in patients and hypothesized that its absence in a mouse model of MI would increase infarct size and adverse remodelling. In patients, LAIR-1 expression was increased 3 days compared to 6 weeks after MI on circulating monocytes (24.8 ± 5.3 vs. 21.2 ± 5.1 MFI, p = 0.008) and neutrophils (12.9 ± 4.7 vs. 10.6 ± 3.1 MFI, p = 0.046). In WT and LAIR-1^−/−^ mice, infarct size after ischemia-reperfusion injury was comparable (37.0 ± 14.5 in WT vs. 39.4 ± 12.2% of the area at risk in LAIR-1^−/−^, p = 0.63). Remodelling after permanent left coronary artery ligation did not differ between WT and LAIR-1^−/−^ mice (end-diastolic volume 133.3 ± 19.3 vs. 132.1 ± 27.9 μL, p = 0.91 and end-systolic volume 112.1 ± 22.2 vs. 106.9 ± 33.5 μL, p = 0.68). Similarly, no differences were observed in inflammatory cell influx or fibrosis. In conclusion, LAIR-1 expression on monocytes and neutrophils is increased in the acute phase after MI in patients, but the absence of LAIR-1 in mice does not influence infarct size, inflammation, fibrosis or adverse cardiac remodelling.

## Introduction

Heart failure (HF) is a complex clinical syndrome and is often caused by reduced cardiac pump function, affecting approximately 1–2% of people in the Western world^1^. The most prevalent cause of HF is an acute myocardial infarction (MI). Despite the significant reduction of early mortality and improved treatment, the prognosis of HF patients remains poor with a 5-year survival of less than 50%^2^, stressing the need for a better understanding and treatment of this complex syndrome.

Heart failure after MI is caused by adverse left ventricular remodelling^3,4^. Adverse remodelling is largely depending on infarct size, but also on the quality of cardiac repair, both greatly influenced by the inflammatory response after MI^5–8^. Although inflammatory cells are important in the clearance of debris and necrotic tissue after MI, their pro-inflammatory activity is also responsible for a variety of detrimental effects. In the (sub)acute phase of ischemia-reperfusion injury, neutrophils and monocytes play an important role in the increase in infarct size^5,9,10^. In the chronic phase, activated and pro-inflammatory monocytes and T-lymphocytes increase adverse remodelling, which leads to impaired cardiac function^11,12^. In both processes, leukocyte activation is key and regulated by integration of signals from activating and inhibitory cell-receptors^13^. The majority of immune inhibitory receptors contain intracellular domains that – upon activation – are able to down regulate or inhibit activation signals from stimulating receptors. Thereby, they increase the threshold for leukocytes to become activated and attenuate pro-inflammatory effects. The transmembrane leukocyte-associated immunoglobulin-like receptor 1 (LAIR-1, CD305) is an inhibitory receptor that is expressed on most cells of the immune system, including natural killer cells, lymphocytes and monocytes^14^. LAIR-1 can inhibit activating signals from ITAM-bearing receptors. Next to this, LAIR-1 is also capable of inhibiting cytokine-mediated signals and it can prevent proliferation and induce apoptosis of human myeloid leukemia cell lines^14^. LAIR-1 is activated upon binding of its ligands including collagens or collagen-domain containing proteins such as surfactant protein D^15,16^. It is counteracted by shedding of its ectodomain (sLAIR-1) or by secretion of its antagonist LAIR-2^17^, which is why plasma levels of these molecules were also studied.

Though LAIR-1 is capable of regulating immune cell function by for example inhibition of target cell lysis or attenuation of the cytotoxic activity of effector T cells^18,19^, its role *in vivo* has been scarcely addressed and evidence for its role in inflammation following acute MI is lacking. Therefore, we compared LAIR-1 expression on leukocytes and circulating levels of sLAIR-1 and LAIR-2 in patients 3 days and 6 weeks after MI, representing the acute and chronic phase of cardiac remodelling. Moreover, we studied the effect of LAIR-1 deficiency in experimental MI in mice, measuring inflammation, infarct size, adverse left ventricular remodelling and cardiac function.

## Materials and Methods

### Study population

Healthy volunteers and patients (>18 years old) with a first time ST-elevation myocardial infarction (STEMI) and non-STEMI from the DEFI-MI (METC: NL45241.041.13) study were included in the current study. Exclusion criteria were the presence of a chronic inflammatory disease, autoimmune disorder, pregnancy and trauma or surgery in the last six months. The Medical Ethics Committee of the UMC Utrecht approved the study and all patients provided written informed consent. The study conforms to the Declaration of Helsinki.

### Patient data collection

**Figure 1:**
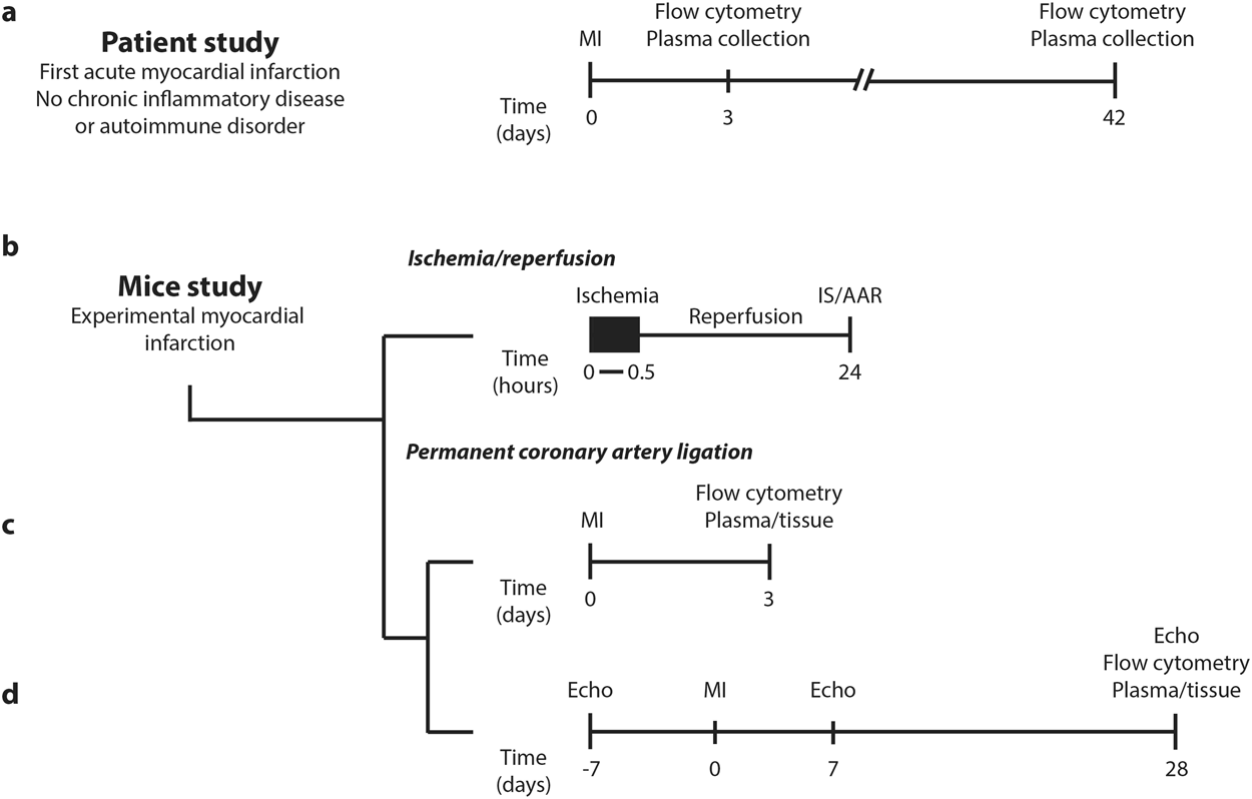
Timeline and experimental set-up. Healthy controls and patients with a first-time MI were included in the current study. Venous blood collection in healthy controls and at 3 days and 6 weeks after MI was used for flow cytometric analyses and to collect plasma (**a**). Mice were either subjected to ischemia-reperfusion injury or to permanent left coronary artery ligation. In the mice subjected to 30 minutes of ischemia and 24 hours of subsequent reperfusion, IS/AAR staining was performed (**b**). In the mice subjected to permanent left coronary artery ligation and sacrificed after 3 days, we performed flow cytometric analyses on various tissues and blood (**c**). In the other mice subjected to left coronary artery ligation, we performed echocardiography at baseline, 7 days and 28 days after MI and performed similar flow cytometric analyses (**d** – lower panel). *MI: myocardial infarction; IS*/*AAR: infarct size*/*area at risk*.

In patients, at the moment of inclusion (3 days after MI) and 6 weeks thereafter, venous blood was drawn and collected by the Laboratory of Clinical Chemistry and Haematology of the UMC Utrecht (Fig. 1a). Whole blood was directly subjected to flow cytometry and EDTA plasma was stored at −80° Celsius in the UMC Utrecht Biobank and used for ELISA (see below). Similarly, blood was drawn from healthy controls and subjected to flow cytometry.

### Animals

Healthy male C57Bl/6 LAIR-1^−/−^^20^ and C57Bl/6 WT littermates (age 10–12 weeks, weight 25–30 g) were housed at room temperature under 12 hour light/dark cycle in groups of maximum 5 animals (in type III cages with filtertop) under strict DM1 regulations and received standard chow and water ad libitum. All animals were genotyped prior to the experimental procedure and animal welfare was monitored daily. A blinded researcher performed surgery on randomly assigned animals (random number generation in excel to animal number, which resulted in alternating fashion of operation of WT and LAIR-1^−/−^ mice). Blinded technicians and observers performed the respective operations, data acquisition and analyses. Sample size calculation for myocardial ischemia reperfusion model was based on infarct size as the primary endpoint. With a power of 90%, alpha of 0.05, estimated effect size of 13% difference in infarct size, standard deviation of 10.4% (based on historical data) and estimated peri-operative mortality of 5% this resulted in a group size of 15 animals per group. For the permanent ligation model sample size calculation was based on end systolic volume as the primary endpoint. With a power of 90%, alpha of 0.05, estimated effect size of 20 μl difference in volume, standard deviation of 15 μl (based on historical data) and estimated peri-operative mortality of 25% this resulted in a group size of 20 animals per group. Cellular influx and collagen content in the ischemic area were defined as secondary outcomes. All animal experiments were approved by the Ethical Committee on Animal Experimentation of the University Medical Center Utrecht (Utrecht, the Netherlands) and conform to the ‘Guide for the care and use of laboratory animals’.

### Induction of myocardial ischemia-reperfusion injury

The experimental set-up and timeline of myocardial ischemia-reperfusion injury is displayed in Fig. 1b. All operations were performed in the morning before noon. In a dedicated mouse operation room, anaesthesia was induced by intraperitoneal (i.p.) injection of medetomidinehydrochloride (1.0 g/kg body weight), midazolam (10.0 mg/kg) and fentanyl (0.1 mg/kg). These anaesthetics were preferred over cardioprotective propofol or volatile anaesthetics (*e.g*. isoflurane)^21^. Mice were intubated and connected to a respirator with a 1:1 oxygen-air ratio (times/minute). A core body temperature of 37 **°**C was maintained during surgery by continuous rectal temperature monitoring and an automatic heating blanket. The heart was accessed through a left lateral thoracotomy with incision of the pericardium. The left coronary artery was ligated for 30 minutes with an 8-0 Ethilon suture (Ethicon) with a section of polyethylene-10 tubing placed over the left coronary artery (LCA). Ischemia was confirmed by bleaching of myocardium and tachycardia. After 30 minutes of ischemia, reperfusion was initiated by releasing the ligation, resulting in tissue colour recurrence. A piece of the suture was left in place to allow for accurate ligature positioning and determination of the ischemic area and the area at risk at termination. The surgical wounds were closed and subcutaneous atipamezole hydrochloride (3.3 mg/kg), flumazenil (0.5 mg/kg) and buprenorphin (0.15 mg/kg) were used as an antagonist. The evening of the day of operation and every 12 hours thereafter, subcutaneous injection of buprenorphin (0.15 mg/kg) was administered as analgesia.

### Infarct size and area at risk quantification after ischemia-reperfusion injury

In total 30 animals (15 WT and 15 LAIR-1^−/−^) were subjected to ischemia-reperfusion injury. Twenty-four hours after ischemia-reperfusion injury, mice were euthanized using sodium pentobarbital (60.0 mg/kg) and a left re-thoracotomy was performed. The LCA was ligated at the same location as it was ligated during index ischemia. The thoracic aorta was cannulated and 2% Evans blue was injected upstream in the aorta to perfuse the coronaries, allowing for staining of the remote but not the area at risk (AAR). The heart was then explanted and rinsed with 0.9% saline to remove superfluous dye. The left ventricle (LV) was dissected and a small piece of gauze was inserted in the left ventricular cavity. After one hour at −20 °C, the LV was cut into 4 equally sized sections. Sections were placed in 1% 2,3,5-triphenyltetrazolium chloride (TTC) in saline and incubated at 37 **°**C for 20 minutes. After 10 minutes, sections were turned to allow for adequate reagent contact. Then, sections were placed in formalin and photographs of both sides of each tissue section were captured using a SZH10 Olympus Zoom Stereo Microscope and IC Capture software, version 2.4. The infarct (white), border zone (red) and remote area (blue) were quantified using ImageJ (version 1.48 v). Infarct size (IS) was expressed as a percentage of the AAR and as a percentage of the LV.

### Induction of myocardial infarction by permanent ligation

Permanent coronary artery ligation was performed as described above for ischemia-reperfusion injury, but leaving the ligature in place, resulting in a permanent occlusion of the left coronary artery. The experimental set-up including the timeline of the mice sacrificed after either 3 days or 28 days is shown in Fig. 1c,d. Surgery was performed on 23 animals (10 WT and 13 LAIR-1^−/−^) for 3 day survival and 38 animals (18 WT and 20 LAIR-1^−/−^) were included for long term survival (28 days).

### Survival

Mice that died after MI were thoroughly inspected for the cause of death. Deaths within 48 hours after MI were considered due to perioperative complications or direct complication of MI. Cardiac rupture was confirmed by massive intrathoracic haemorrhage >48 hours after operation and ventricular leakage of the myocardium upon perfusion of the heart with 0.9% saline. In the ischemia reperfusion model 2 LAIR-1^−/−^ died due to perioperative conditions. Of the animals exposed to 3 days permanent ligation 1 WT animal died during the surgical procedure, of animals exposed to 28 days permanent ligation 6 WT and 9 LAIT-1^−/−^ mice died. Of the 6 WT, 5 died due to cardiac rupture and 1 to unkown causes. Of the LAIR-1^−/−^ 8 died due to cardiac ruputre and 1 to unknown causes.

### Echocardiography

At baseline, 7 and 28 days after permanent ligation, anaesthesia was induced by inhalation of 2.0% isoflurane in a mixture of oxygen/air (1:1). Echocardiography was used to assess cardiac geometry and function. Heart rate, respiration and rectal temperature were constantly monitored and body temperature was kept between 36.0 and 38.0 °C using heat lamps. Respiration gating, a 3-dimensional motor and trigger points were used to obtain 300 transversal images of the heart during the expiratory phase, either at the end of systole or the end of diastole. These images were then used for complete 3D reconstruction of the heart. Image acquisition and analyses were performed using the dedicated Vevo® 2100 System and Software (Fujifilm VisualSonics Inc., Toronto, Canada).

### Tissue processing and histological analyses

At the end of the follow-up period, mice were euthanized using sodium pentobarbital (60.0 g/kg). Blood was collected through orbital puncture in EDTA tubes. The inferior caval vein was incised and the vascular system was flushed with 5 mL phosphate-buffered saline (PBS) through right ventricular puncture.

The spleen and the mediastinal lymph nodes posterior to the heart were excised and contained in PBS for flow cytometric analyses afterwards. Then, the heart was explanted and cut in half. One half was dissected further into infarct and remote tissue and used for flow cytometry or snap-frozen in liquid nitrogen. The other half was formalin-fixed for 24 hours, embedded in paraffin and cut into 5 μm thick sections. Neutrophils were stained using a rat monoclonal mouse LY-6G (GR-1) antibody (1:400, Biolegend, 108402, 0.5 mg/ml). Rabbit-anti-rat-biotin (1:200, DAKO E0468, 0.84 g/L) was used as a secondary antibody and Streptavidin-AP (1:500, SA-5100) as a tertiary antibody. T-cells were stained using a polyclonal rabbit-anti-human CD3 antibody (1:100, Dako, A0452) and anti-rabbit-AP Powervision (pure, DPVR-110AP, Immunologic) was used as secondary antibody. Macrophages were stained using a rat-anti-mouse MAC3 antibody (1:30, BD Pharmingen, 553322, 0.5 mg/ml). Rabbit-anti-rat-biotin (1:200, DAKO E0468, 0.84 g/L) was used as a secondary antibody and Streptavidin-AP (1:500, SA-5100) as a tertiary antibody. Liquid permanent red was used as an enzyme substrate. The different subtypes of collagen were stained using goat-anti type I Collagen (1:250, Southern Biotech, 1310-01, 0.4 mg/ml), goat-anti type III Collagen (1:50, Southern Biotech, 1330-01, 0.4 mg/ml) or goat-anti type IV Collagen (1:100, Southern Biotech, 1340-01, 0.4 mg/ml) and alexa 488 donkey-anti-goat (1:250, Invitrogen, A11055, 2 mg/ml) as a secondary antibody.

Neutrophils, T-cells, macrophages and collagens were semi-automatically quantified using digital histology. Collagen content was quantified in tissue sections stained for picrosirius red and photographed under polarized light, converted to gray scale images and expressed as a percentage of the region of interest (*i.e*. infarct, remote). Images of tissue sections were captured and analysed using CellSens (Olympus Corporation, Tokyo, Japan). Of the 9 WT and 10 LAIR-1^−/−^) mice at 3 days follow-up, 1 WT and 1 LAIR^−/−^ mice were excluded for histological analysis due to the absence of a clearly identifiable infarction. For neutrophil analysis an 2 WT and 2 LAIR-1^−/−^) animals were excluded due to technical errors. Of the 11 WT and 10 LAIR1^−/−^ mice at 28 days follow-up 1 WT and 1 LAIR-1^−/−^) mice were excluded for histological analysis due to the absence of a clearly identifiable infarction. For macrophage analysis an 3 LAIR-1^−/−^) animals were excluded due to technical errors. 2. Technical errors may occur due to poor quality upon sectioning or inferior quality of immunohistochemistry stainings, which makes reliable analysis impossible.

### Flow cytometric assays

Fresh human EDTA blood (50 μL) was added to an antibody mixture containing different cell surface markers to identify neutrophils and monocytes (see Supplementary Table S1). Cells were incubated for 30 minutes in the dark at room temperature (RT). Before measurement, cells were washed and erythrocytes were lysed using Optilyse C.

To harvest single cells from heart tissue, enzymatic degradation was performed (N = 6 WT and N = 6 LAIR-1^−/−^). Infarct and remote tissue were collected 3 days after MI and cut into small pieces of around 1 mm^2^. Dissociation solution (10 × 10^2^ U/ml DNase I (Roche 04536282001), 10 mM HEPES (Life Technology 15630-080) and 2.6 U/ml Liberase TL (Roche 05401020001)) was added to the tissue and incubated at 37° Celsius for 20 minutes. Single cells of the dissociated myocardial tissue, lymph nodes and spleen were obtained through gentle filtering over a 40 µm cell strainer and subsequently incubated with an antibody mixture containing different cell surface markers to identify neutrophils, monocytes, and T- and B-lymphocytes (see Supplementary Table S2) for 30 minutes in the dark at RT. After washing, residual red blood cells were lysed with erythrocyte-lysis buffer. All samples were measured on a Gallios flow cytometer (10 colour configuration, Beckman Coulter, Marseille, France). Kaluza Analysis Software 1.3 was used for data analysis. The gating strategy is shown in Supplementary Fig. S1.

### ELISA

Plasma levels of soluble LAIR-1 (sLAIR-1) and LAIR-2 were measured in duplo using a respective sLAIR-1 and LAIR-2 sandwich ELISA according to manufacturer’s instructions (LifeSpan BioSciences, Seatle, WA, USA). Colorimetric analyses were performed using a spectrophotometer (450 nm). Plasma levels were calculated based on standards.

### High-sensitivity multiplex immunoassay

Plasma levels of IL-6 and TNFα were measured in duplo using a high-sensitivity ProcartaPlex multiplex immunoassay according to manufacturer’s instructions (EPXS010-20603-901 and EPXS010-20607-901, ThermoFisher Scientific). Plasma levels were calculated based on standards. Il-6 and TNFα values were ln-transformed.

### Statistical analyses

Data distribution was evaluated for normality using the d’Agostino & Pearson normality test. Data are expressed as mean ± standard deviation (SD). Skewed ELISA and immunoassay data were ln-transformed and presented as median with interquartile range (IQR). Normally distributed data were compared using a two-tailed paired (serial measurements) or unpaired t-test (separate groups). Non-normally distributed data were compared using a Wilcoxon (serial measurements) or Mann-Whitney test (separate groups). A log-rank (Mantel-Cox) test was used for survival analysis. A level of p < 0.05 was considered statistically significant. Statistical analyses were performed using SPSS software, version 21 and GraphPad Prism, version 6.

## Results

### In patients, LAIR-1 expression on circulating cells and sLAIR-1 and LAIR-2 plasma levels differ between the acute and chronic phase after MI

**Figure 2:**
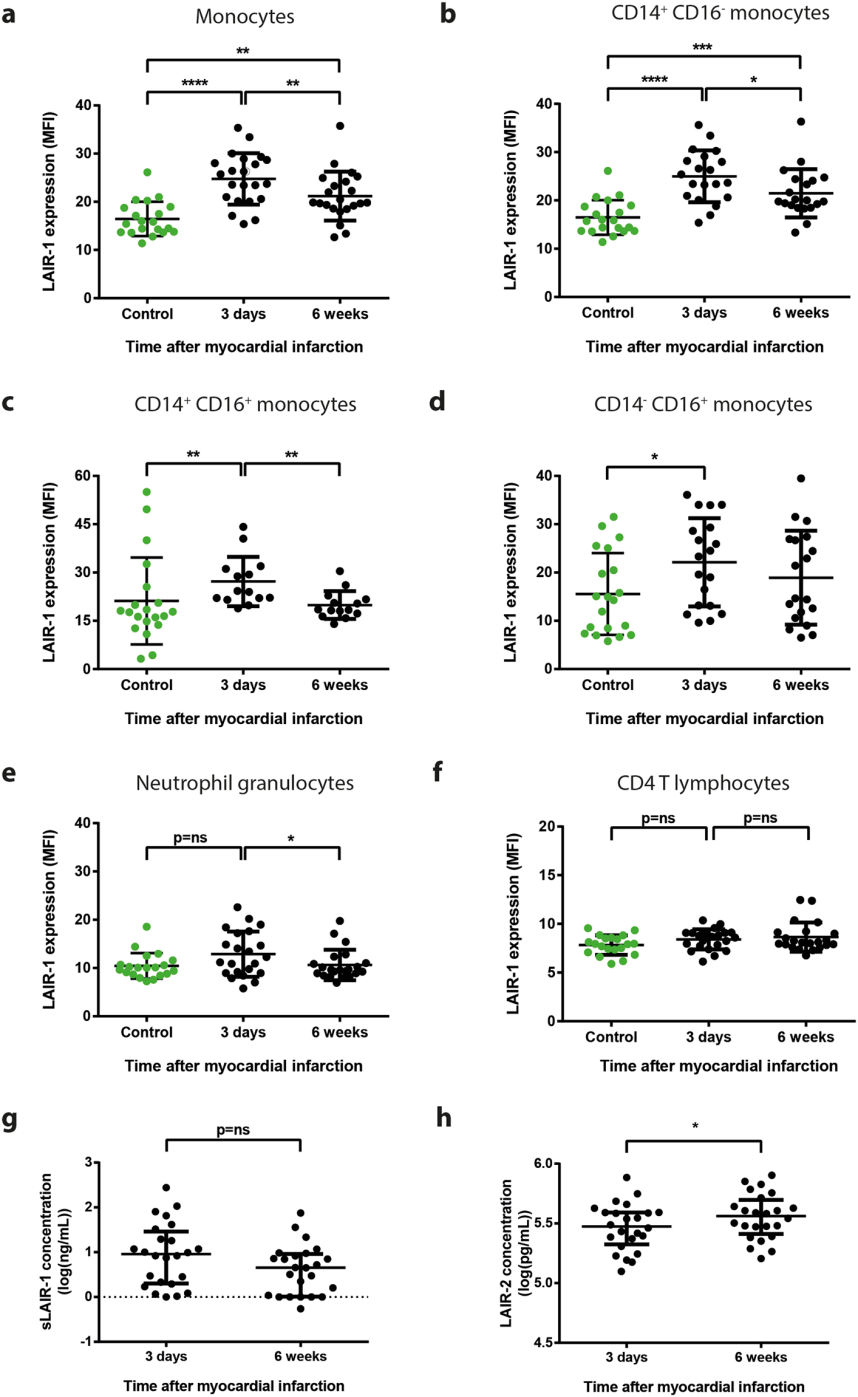
LAIR-1 expression on leukocyte subsets and sLAIR-1 and LAIR-2 plasma levels differ between the acute and chronic phase after myocardial infarction in patients. Flow cytometry showed that LAIR-1 receptor expression on monocytes (**a**) 3 days after MI was higher than 6 weeks thereafter and compared to healthy controls, which could be mainly attributed to CD14^++^ CD16^−^ classical (**b**) and CD14^++^ CD16^+^ intermediate monocytes (**c**), but not to CD14^−^ CD16^+^ non-classical monocytes (**d**). Similar to monocytes, also granulocytes showed higher LAIR-1 receptor expression in the acute compared to the chronic phase, but no difference between the chronic phase and healthy controls was observed (**e**). No difference was observed in LAIR-1 receptor expression on CD4^+^ T-lymphocytes (**f**). Though not significant, sLAIR-1 was higher 3 days after MI compared to 6 weeks (**g**). In contrast, plasma levels of LAIR-2 were lower 3 days after MI compared to 6 weeks (**h**). N = 19–22 (**a,b**,**d,e**), 14 (**c**) and 24 (**f,g**) patients, 20 healthy controls (**a**–**e**). *MI: myocardial infarction;* *p < 0.05, **p < 0.01, ***p < 0.001, ****p < *0.0001*.

Out of 24 patients included in this study, 22 patients (92%) suffered from a STEMI and 2 patients (8%) from a non-STEMI (Table 1). The mean age was 58 ± 11 years and the majority of patients were male (79%). For comparison, 20 healthy volunteers were included as controls. The transmembrane expression of LAIR-1 on monocytes was significantly higher in the acute phase (3 days after MI), compared to the chronic phase (24.8 ± 5.3 at day 3 vs. 21.2 ± 5.1 MFI at 6 weeks post MI, p = 0.008; Fig. 2a), and both were significantly increased with respect to healthy controls. Subgroup analyses showed that this difference could be attributed to higher LAIR-1 expression on pro-inflammatory CD14^++^ CD16^−^ classical (25.0 ± 5.4 at 3 days vs. 21.5 ± 5.0 MFI at 6 weeks post MI, p = 0.013; Fig. 2b) and CD14^++^ CD16^+^ intermediate monocytes (27.2 ± 7.7 at day 3 vs. 19.9 ± 4.3 MFI at 6 weeks post MI, p = 0.001; Fig. 2c), but not CD14^−^ CD16^+^ non-classical monocytes (22.1 ± 9.1 at day 3 vs. 18.9 ± 9.7 MFI at 6 week, p = 0.28; Fig. 2d). Similar to monocytes, LAIR-1 expression on neutrophils was higher 3 days after MI compared to 6 weeks after MI (12.9 ± 4.7 vs. 10.6 ± 3.1 MFI, p = 0.046; Fig. 2e). There was no difference in LAIR-1 expression on CD4^+^ T-lymphocytes (8.4 ± 1.0 at day 3 vs. 8.7 ± 1.5 MFI at 6 weeks, p = 0.95; Fig. 2f) or CD8^+^ T-lymphocytes (10.6 ± 3.0 at day 3 vs 11.0 ± 3.5 MFI at 6 weeks, p = 0.92; see Supplementary Fig. S3).

**Table 1:** Baseline characteristics of DEFI-MI patients.

DEFI-MI patients (N = 24)	
Male sex	19 (79%)
Age	58 ± 11 years
**Cardiovascular risk factors**	
BMI	25.5 ± 2.4
Diabetes	0 (0%)
Hypertension	5 (21%)
Hypercholesterolemia	8 (33%)
Smoking	8 (33%)
**Medication use**	
Aspirin	20 (83%)
P2Y12 inhibitor	24 (100%)
Statin	23 (96%)
Beta-blocker	20 (83%)
RAAS-inhibitor	22 (92%)
**History of cardiovascular disease**	
CVA/TIA	0 (0%)
Peripheral artery disease	0 (0%)
Chronic kidney failure	0 (0%)
**Indication**	
STEMI	22 (92%)
Non-STEMI	2 (8%)

Clinical characteristics of DEFI-MI patients upon presentation. BMI: body mass index; RAS: renin-angiotensin system; CVA: cerebrovascular accident; TIA: transient ischemic attack; STEMI: ST-elevation myocardial infarction.

In general, comparison with healthy controls showed that LAIR-1 expression was increased after MI on monocytes and neutrophils, either at both 3 days and 6 weeks (monocytes, CD14^++^ CD16^−^ classical monocytes) or at 3 days only (CD14^++^ CD16^+^ intermediate monocytes, CD14^−^ CD16^+^ non-classical monocytes).

Plasma levels of sLAIR-1 were slightly higher 3 days after MI compared to 6 weeks, though this was not significant (2.71 IQR [1.35–3.87] vs. 1.92 IQR [1.01–2.61], p = 0.07; Fig. 2g). In contrast, LAIR-2 levels were significantly higher after 6 weeks (238.7 IQR [205.2–268.0] vs. 260.3 IQR [223.9–297.7], p = 0.049; Fig. 2h).

### The absence of LAIR-1 does not influence infarct size in mice

**Figure 3:**
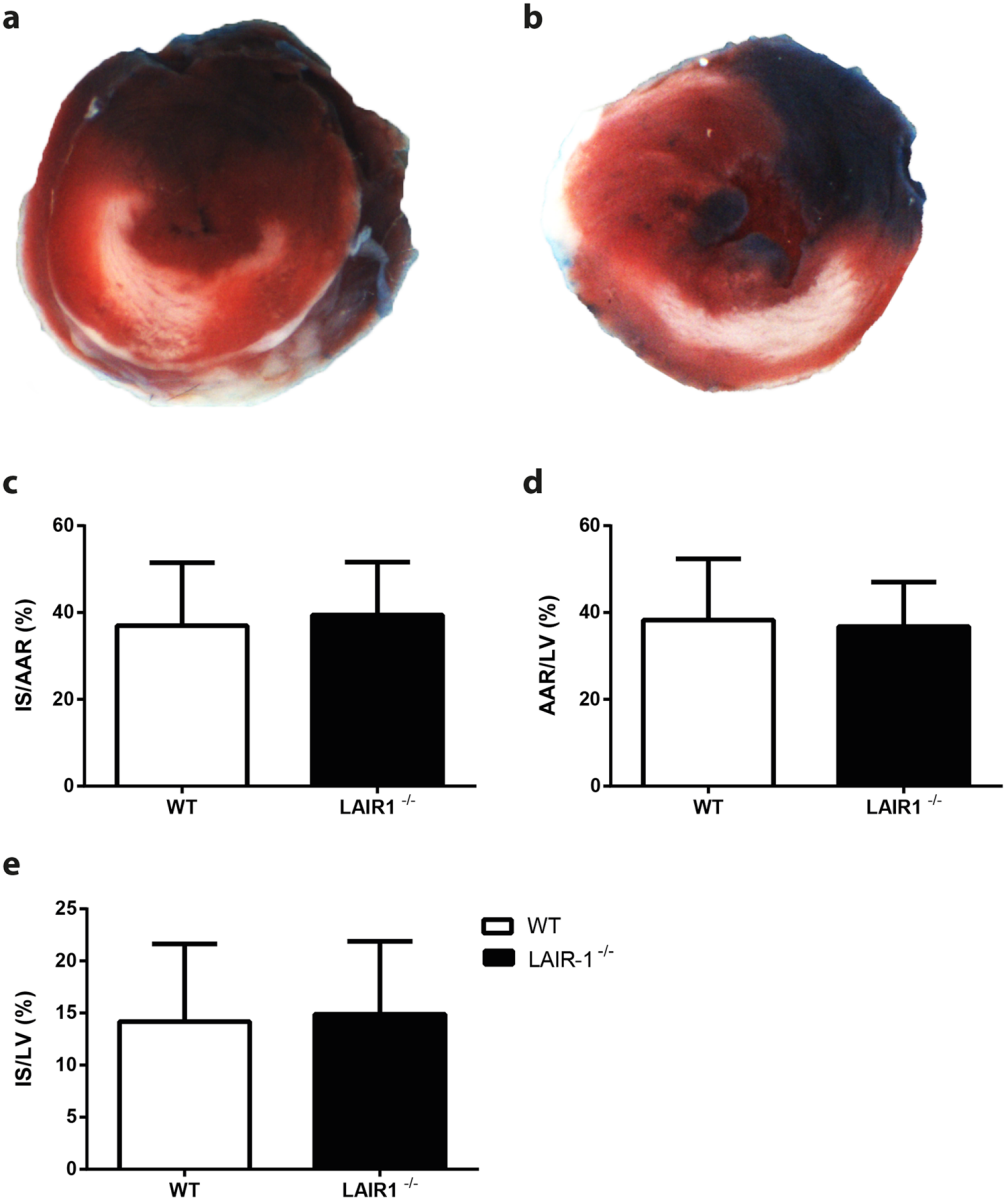
Infarct size and area at risk quantification after ischemia-reperfusion injury in mice. Evans blue and TTC staining of WT (**a**) and LAIR-1^−/−^ hearts (**b**) were used for the quantification of infarct size (IS; white), area at risk (AAR; sum of white and red area) and the left ventricle itself (LV; entire area). Ischemia-reperfusion injury assessed through IS/AAR% did not differ between both groups (**c**). Also AAR/LV% (**d**) and IS/LV% was comparable between both groups. N = 15 WT and 13 LAIR-1^−/−^ animals per group. *WT: wild-type; LAIR-1* ^−/−^ *: LAIR-1 deficient*.

Evans blue and TTC staining were used to quantify infarct size (IS), area at risk (AAR) and left ventricular (LV) area in both WT and LAIR-1^−/−^ mice (Fig. 3a,b). Ischemia-reperfusion injury as assessed by IS/AAR did not differ between WT and LAIR-1^−/−^ mice (37.0 ± 14.5 vs. 39.4 ± 12.2%, p = 0.63; Fig. 3c). In addition, AAR/LV was comparable between both groups (38.3 ± 14.1 vs. 36.8 ± 10.3%, p = 0.75; Fig. 3d), as was IS/LV (14.2 ± 7.4 vs. 14.9 ± 7.0, p = 0.80; Fig. 3e).

### Survival, cardiac geometry, and cardiac function are comparable between wild-type and LAIR-1^−/−^ mice after permanent coronary artery ligation

**Figure 4:**
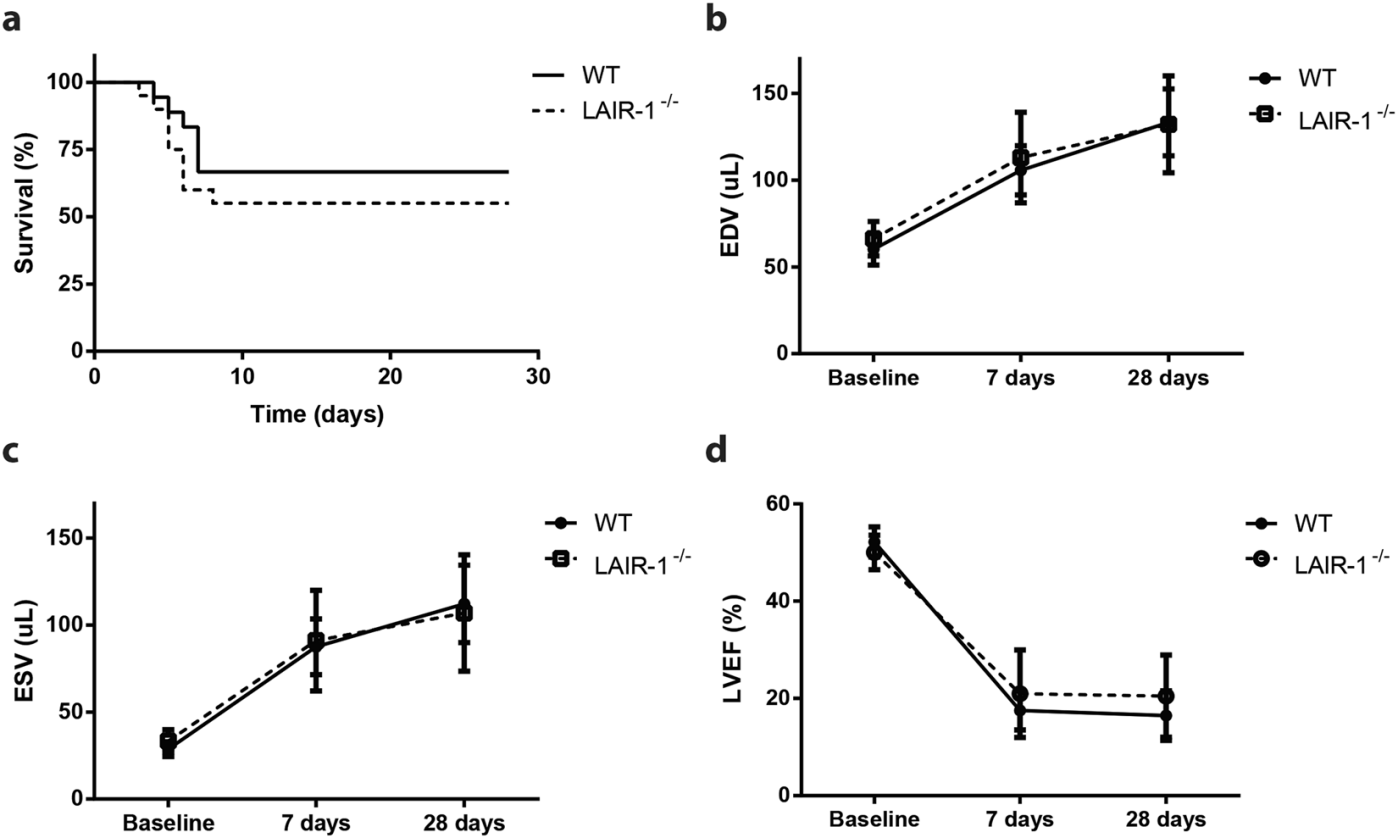
Survival and geometric dimensions by 3D echocardiography in mice. Within the follow-up period of 28 days after permanent occlusion of the left coronary artery, 6 out of 18 WT mice and 9 out of 20 LAIR-1^−/−^ mice died as a consequence of cardiac rupture (**a**). In the surviving animals, EDV, ESV and LVEF were comparable 7 and 28 days after ligation (**b**–**d**). N = 12 WT and 11 LAIR-1^−/−^ surviving animals per group. *WT: wild-type; LAIR* ^*−*/*−*^ *: LAIR-1 deficient; EDV: end-diastolic volume; ESV: end-systolic volume; LVEF: left ventricular ejection fraction*.

Within 28 days after permanent ligation, cardiac rupture and subsequent death occurred in 6 out of 18 WT and 9 out of 20 LAIR-1^−/−^ mice (33.3 vs. 45.0%, p = 0.38; Fig. 4a). In the surviving animals, no differences were observed with respect to end-diastolic volume (EDV) at 7 days (WT 105.6 ± 14.3 vs. LAIR-1^−/−^ 113.0 ± 26.1 μL, p = 0.40; Fig. 4b) and 28 days (133.3 ± 19.3 vs. 132.1 ± 27.9 μL, p = 0.91) after permanent ligation. In addition, end-systolic volume (ESV) was comparable between both groups after 7 days (87.5 ± 16.1 vs. 91.0 ± 29.0 μL, p = 0.73; Fig. 4c) and 28 days (112.1 ± 22.2 vs. 106.9 ± 33.5 μL, p = 0.68). Correspondingly, left ventricular ejection fraction did not differ between WT and LAIR-1^−/−^ mice at 7 days (17.6 ± 4.0 vs. 21.0 ± 9.0%, p = 0.25; Fig. 4d) and 28 days (16.5 ± 5.1 vs. 20.5 ± 8.5%; p = 0.20) after MI.

### Wild-type and LAIR^−/−^ mice show no differences in inflammatory responses following myocardial infarction

**Figure 5:**
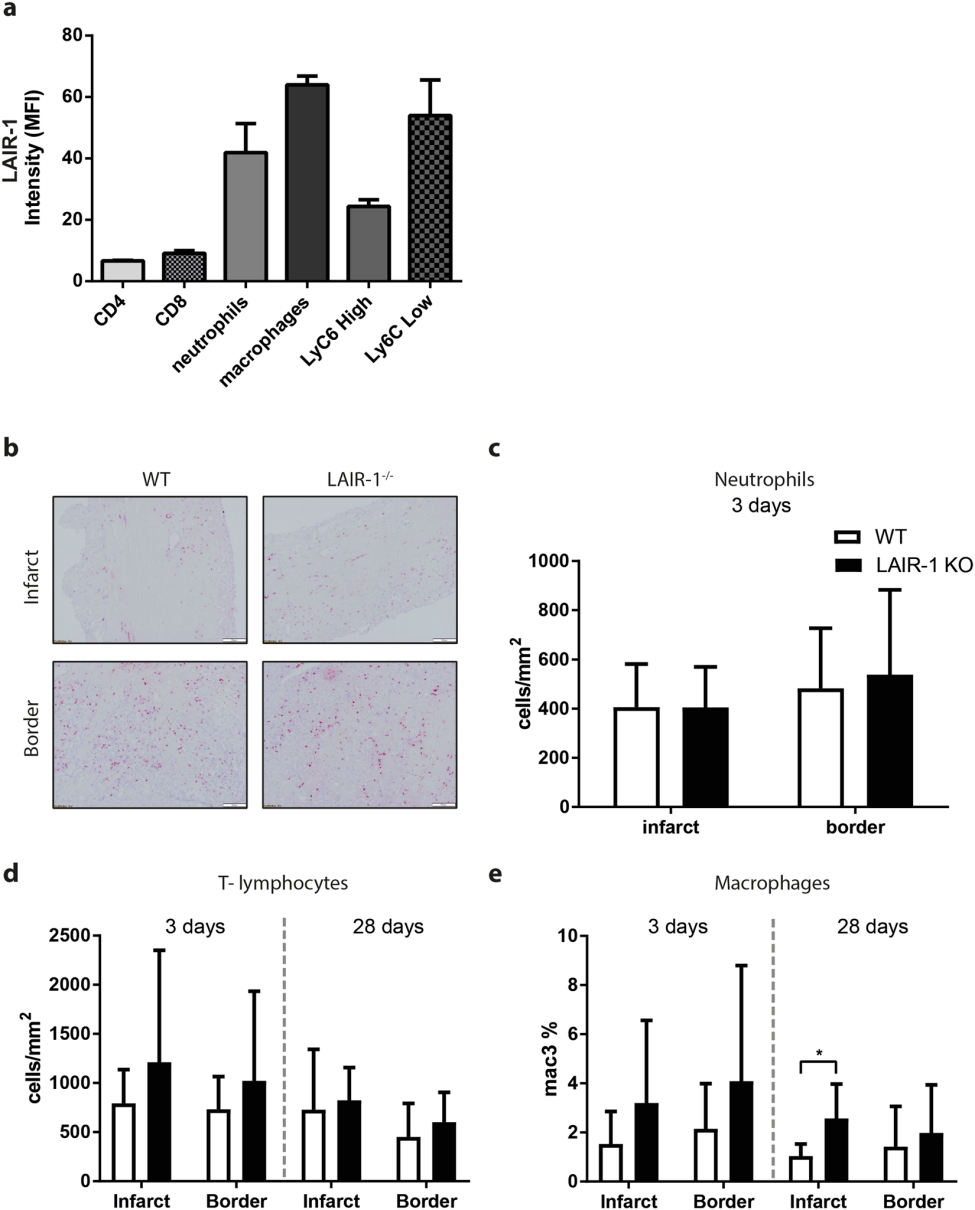
Leukocytes levels in circulation and infiltration in the murine heart. LAIR-1 expression on different leukocyte subset in peripheral blood from baseline WT mice was determined by flow cytometry (N = 9 animals). LAIR-1 expression was most pronounced on cells of myeloid origin, but also observed on CD4 and CD8 T-lymphocytes (**a**). To assess neutrophil infiltration in the infarcted murine myocardium, cardiac tissue sections were stained with Ly6G three days after MI. Representative images of WT and LAIR-1^−/−^ mice of the infarct area and border zone (**b**) showed no difference in neutrophil influx three days after permanent occlusion of the left coronary artery (N = 6 WT and 7 LAIR-1^−/−^) (**c**). Cardiac T-lymphocyte influx was not different three or 28 days after permanent occlusion of the left coronary artery (N = 8 WT and 9 LAIR-1^−/−^) (N = 11 WT and 10 LAIR-1^−/−^) (**d**). Macrophage influx on showed a difference between WT and LAIR-1^−/−^ mice in the infarct zone 28 days after MI (**e**). Scale bar 100 µm. *WT: wild-type; LAIR-1* ^−/−^ *: LAIR-1 deficient*. *p < *0.05*.

To confirm LAIR-1 expression on circulating leukocytes, we performed flow cytometry on baseline blood. In WT mice, LAIR-1 was expressed on CD4^+^ T-cells and CD8^+^ T- cells, but most prominent on cells of myeloid origin, amongst which neutrophils, macrophages and Ly6C expressing monocytes (Fig. 5a). While LAIR-1 expression was similar over time in T-cells and monocytes, we did observe a decrease in LAIR-1 expression on macrophages 3 days and on neutrophils 28 days after MI (Supplementary Fig. S4). As expected, LAIR-1 was undetectable on cells from LAIR-1^−/−^ mice.

Flow cytometry was performed for the characterization and quantification of leukocytes in the blood, spleen, draining lymph node and heart after MI (see Supplementary Fig. S2). Three days after MI, a robust leukocyte influx in the heart was observed, that mostly consisted of neutrophils and CD8^+^ T-cells (Table 2). No difference in white blood cell subtype composition was observed between WT and LAIR-1^−/−^ mice 3 days and 28 days after MI in all studied organs (Table 2 – blood, infarct area; see Supplementary Table S3-S4 – remote area, lymph nodes, spleen).

**Table 2:** Leukocyte levels in blood and infarct area after 3 days of MI.

	Blood		Infarcted myocardium	
	WT	LAIR-1^−/−^	WT	LAIR-1^−/−^
Neutrophils	28.1 ± 23.5%	22.6 ± 8.3%	56.8 ± 11.9%	49.4 ± 14.3%
Macrophages	5.4 ± 4.4%	7.1 ± 4.0%	25.9 ± 8.2%	30.9 ± 8.7%
Ly6C High monocytes	2.0 ± 1.5%	3.0 ± 2.0%	3.0 ± 1.9%	4.5 ± 1.8%
Ly6C Low monocytes	4.5 ± 0.6%	5.0 ± 1.2%	3.9 ± 2.4%	4.6 ± 2.9%
CD4 T-cells	9.0 ± 2.6%	11.4 ± 3.2%	1.9 ± 1.1%	2.0 ± 1.2%
CD8 T-cells	8.7 ± 3.8%	10.9 ± 2.5%	34.3 ± 6.8%	42.5 ± 10%

Leukocyte levels in blood and infarct area three days after myocardial infarction in mice. Percentages are given of total leukocytes. N = 6 WT and 5 LAIR-1^−/−^.

Leukocyte activation after MI increases the release of various cytokines, of which IL-6 and TNFα are well known. Using a high-sensitive multiplex immunoassay, we compared plasma cytokine levels in WT and LAIR-1^−/−^ mice. TNFα levels were borderline detectable, did not change after MI and were comparable in both groups (Supplementary Fig. S4). In contrast to stable IL-6 levels in WT mice, IL-6 did increase in LAIR-1^−/−^ mice 3 days after MI (25.7 IQR 25.6 – 25.8 vs. 437 IQR 166.7–586.5, p = 0.04). A subsequent decrease in IL-6 levels 28 days after MI was observed in both WT and LAIR-1^−/−^ mice (Supplementary Fig. S6). Overall, IL-6 levels were not significantly different between WT and LAIR1^−/−^ mice.

Since neutrophils are the first to infiltrate the heart in the early phase after MI and are notorious for the additional damage they cause to the myocardium, we performed a neutrophil staining. Three days after MI, neutrophil influx was comparable between WT and LAIR-1^−/−^ mice in both the infarct area (406 ± 167 vs. 405 ± 165 cells/mm^2^, p = 0.99; Fig. 5b,c) and border zone (483 ± 245 vs. 539 ± 344 cells/mm^2^, p = 0.63). In addition, T-cells and macrophages play an important role in remodelling after MI. CD3^+^ staining showed a comparable infiltration of T-cells in the myocardium of WT and LAIR-1^−/−^ mice both in the infarct area (792 ± 343 vs. 1212 ± 1139 cells/mm^2^, p = 0.33; Fig. 5d) and border zone (733 ± 333 vs. 1021 ± 913 cells/mm^2^, p = 0.41) 3 days after MI. Similar results were observed twenty-eight days after MI in the infarct area (728 ± 616 vs. 824 ± 335 cells/mm^2^, p = 0.67; Fig. 5d) and border zone (450 ± 344 vs. 601 ± 304 cells/mm^2^, p = 0.30). Also, macrophage infiltration did not differ between WT and LAIR-1^−/−^ mice 3 days after MI in both the infarct area (1.5 ± 1.3 vs. 3.2 ± 3.4% positive area, p = 0.20; Fig. 5e) and border zone (2.1 ± 1.8 vs. 4.1 ± 4.7% positive area, p = 0.28). After 28 days however, fewer macrophages were present in the infarct area of WT mice compared to LAIR-1^−/−^ mice (1.0 ± 0.5 vs. 2.6 ± 1.4% positive area, p = 0.01; Fig. 5e) but this was not the case in the border zone (1.4 ± 1.6 vs. 2.0 ± 2.0% positive area, p = 0.54).

### Fibrosis formation is not affected by the absence of LAIR-1^−/−^ after chronic myocardial infarction

**Figure 6:**
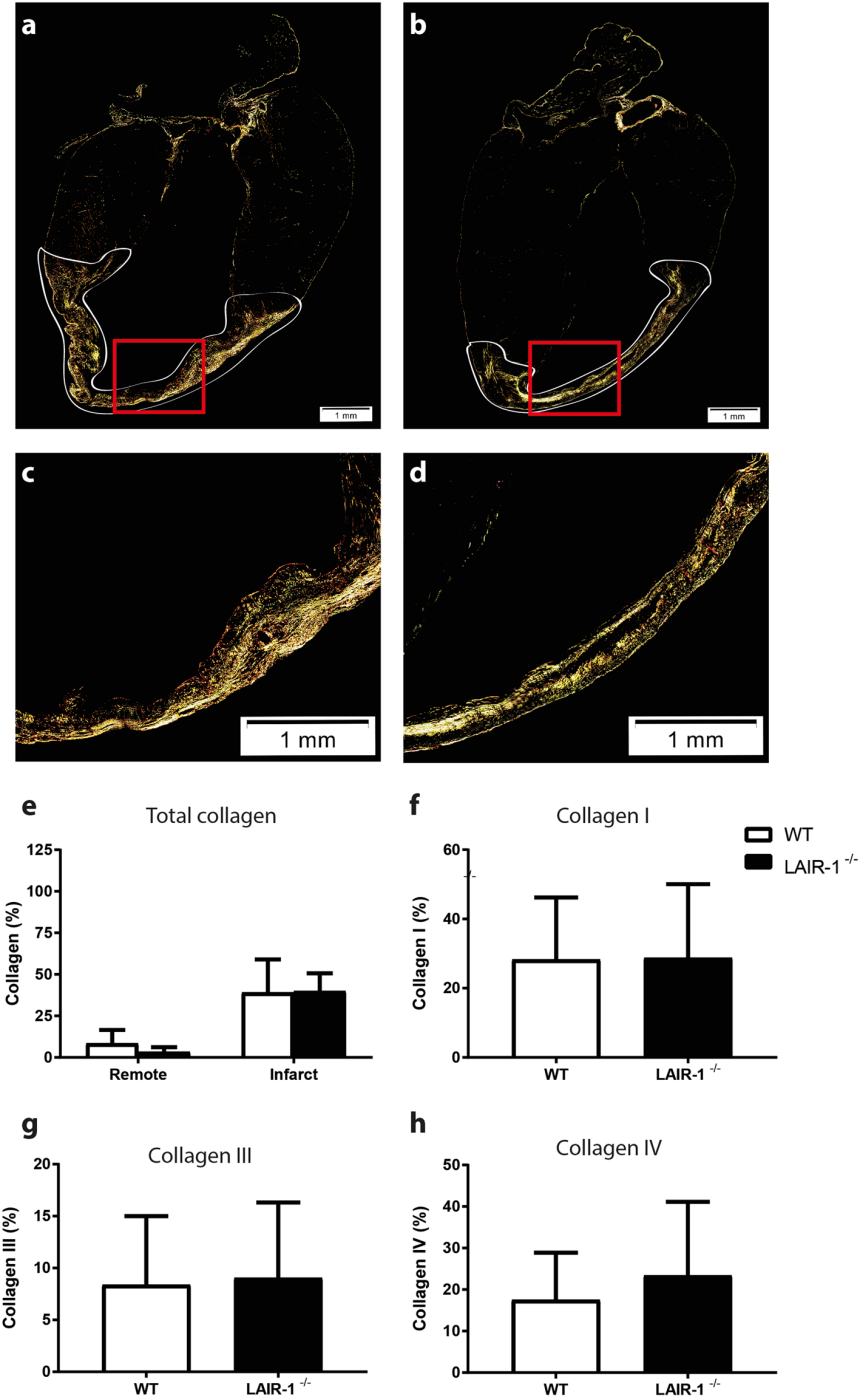
Collagen content 28 days after permanent coronary artery ligation. Cardiac tissue sections were stained with picrosirius red and photographed under polarized light. Representative overview images of WT and LAIR-1^−/−^ mice (**a**,**b**) and magnified images of the infarct area (**c**,**d**) show no differences in total collagen percentage in both remote and infarct regions 28 days after permanent occlusion of the left coronary artery (**e**). There was also no difference in the percentage of collagen subtypes I (**f**), III (**g**) or IV (**h**). N = 11 WT and 10 LAIR-1^−/−^ animals per group. Scale bar 100 µm. *WT: wild-type; LAIR-1* ^−/−^ *: LAIR-1 deficient*.

As a marker of cardiac fibrosis we stained cardiac tissue sections with picrosirius red of both WT and LAIR-1^−/−^ mice (representative pictures in Fig. 6a,b). Magnified images of the infarct area (Fig. 6c,d) show no differences in collagen content between both groups 28 days after permanent ligation (39.1 ± 19.9 vs. 40.0 ± 10.7%, p = 0.65; Fig. 6e). In addition, different collagen types were distinguished using specific a staining for collagen I, collagen III and collagen IV, respectively. Infarct tissue consists mainly of Collagen I (WT: 28.3 ± 17.9, LAIR-1^−/−^: 28.8 ± 21.3%, p = 0.97) and Collagen IV (WT: 17.5 ± 11.4, LAIR-1^−/−^: 23.4 ± 17.8%, p = 0.60), whereas collagen III comprised about 8% of the infarct area (WT: 8.4 ± 6.6, LAIR-1^−/−^: 9.0 ± 7.3%, p = 0.97). No difference in either collagen type was observed between WT and LAIR-1^−/−^ mice (Fig. 6f–h).

## Discussion

Leukocytes and leukocyte activation, in particular monocytes and neutrophils, have been shown to play an important role in both cardiac ischemia-reperfusion injury and remodelling^22–25^. LAIR-1 is present on a variety of immune cells^14^ and important in the regulation of leukocyte activation in response to an inflammatory reaction^15,26,27^. We observed increased LAIR-1 expression on leukocytes of patients compared to healthy controls. More specifically, LAIR-1 expression on circulating monocytes and neutrophils is increased directly after MI and declines after six weeks, suggestive of immune regulation by LAIR-1 in a response to the pro-inflammatory environment of MI. In more detail, LAIR-1 expression differed on pro-inflammatory CD14^++^ CD16^−^ classical and CD14^++^ CD16^+^ intermediate monocytes. Although both are necessary for the removal of debris following MI, their effect is generally considered disproportionate and detrimental^11^. Therefore, higher LAIR-1 expression in the acute phase after MI may be beneficial in suppressing pro-inflammatory monocyte activation to limit cardiac damage. Although LAIR-1 expression on monocytes decreases in the chronic phase after MI, the expression levels remain increased when compared to healthy controls. This is most probably linked to ongoing low-grade inflammatory response in the chronic phase of cardiac remodelling after MI^28^. In addition, we observed higher LAIR-1 expression on neutrophils in the acute phase after MI compared to the chronic phase. Considering the observation that pro-inflammatory stimuli lead to the higher LAIR-1 expression on neutrophils^29^, this is in agreement with the strong inflammatory response directly after MI.

Next to increased LAIR-1 expression on monocytes and neutrophils, we also observed higher levels of sLAIR-1 in the acute phase after MI, which is in line with the observation that cell activation induces shedding of LAIR-1^30^. Although the source of sLAIR-1 remains to be elucidated, this finding suggests that inflammation in the acute setting of MI increases LAIR-1 expression even more to result in both high expression levels and a high amount of LAIR-1 shedding. Contrarily, the levels of LAIR-2, mainly produced by stimulated CD4^+^ T-lymphocytes, are lower in the acute phase of MI. This difference might be (partially) explained by the relatively decreased number of circulating CD4^+^ T-cells in the acute stage after MI compared to the chronic phase^31^. Though both sLAIR-1 and LAIR-2 are natural antagonists of cell-bound LAIR-1, LAIR-2 has been shown to be far more potent than sLAIR-1.

These findings in patients prompted us to study if LAIR-1 is causally involved in ischemia reperfusion injury in the heart. However, in mice, the absence of LAIR-1, did not affect infarct size or cardiac remodelling after MI. Although leukocyte activating receptors and inflammation are widely recognized as important players in ischemia-reperfusion injury and remodelling after MI^32–34^, and despite the regulation in LAIR expression in MI patients, we were not able to establish a causal role for LAIR-1 deficiency in this regard.

The extent of ischemia-reperfusion injury is in agreement with previously performed experiments in WT mice in our laboratory^34–36^, infarct size after myocardial ischemia-reperfusion did not differ between WT and LAIR-1^−/−^ mice. We anticipated on increased reperfusion injury in the LAIR-1^−/−^ mice as a consequence of enhanced cellular infiltration and inflammation. However, the inflammatory response assessed in various tissues in the acute (3 days) and more chronic (28 days) inflammatory phase after MI did not differ between WT and LAIR-1^−/−^ mice. In addition, the deposition of collagen, as well as the extent of cardiac remodelling at 28 days was comparable to those observed in previously performed experiments^37,38^, but did not differ between both groups.

Activating leukocyte receptors^34,39,40^ and costimulatory molecules^41^ have been shown to play an important role in myocardial reperfusion injury through modulation of the inflammatory response, whereas studies on inhibitory receptors or co-inhibitory molecules are lacking. The inhibitory effect of LAIR-1 may not provide sufficient potency for the extent of tissue damage and severity of the inflammatory response in the present model, as was previously shown *in vitro* ^42^. This is in agreement with the observation that LAIR-1 has been shown to be primarily involved in low-grade chronic inflammatory diseases, such as cancer^19,43^ and chronic contact dermatitis^44^, but not so much in acute, high grade, inflammatory responses as observed in experimental autoimmune encephalitis and LPS injection^45^. Although the chronic phase of myocardial remodelling shows a somewhat less inflammatory response than the acute phase of experimental MI, leukocyte influx is still impressive^46^.

In addition, cell activation starts in the bloodstream^47^, whereas inhibition of LAIR-1 is expected to occur predominantly upon the encounter of collagen in the heart. This may either be too late to efficiently inhibit the already initiated pro-inflammatory cascade, or the sheer amount of collagen ligands is too low to induce robust activation of LAIR-1. Moreover, other inhibitory receptors and/or pathways could have compensated for the absence of LAIR-1.

In conclusion, LAIR-1 expression on monocytes and neutrophils is increased in patients 3 days after MI. Though, in mice, the absence of LAIR-1 does not influence infarct size, nor does it affect inflammation, fibrosis formation and adverse left ventricular remodelling in mice four weeks after acute MI.

## Electronic supplementary material

Supplementary data
