# Supplementary material for: Leukocyte-Associated Immunoglobulin-like Receptor-1 is regulated in human myocardial infarction but its absence does not affect infarct size in mice
Source: Sci Rep. 2017 Dec 21;7:18039. doi: 10.1038/s41598-017-13678-5 (PMC5740066; doi:10.1038/s41598-017-13678-5)
Supplement: Supplementary file 1 — Supplementary data [file 41598_2017_13678_MOESM1_ESM.doc]

**Leukocyte-Associated Immunoglobulin-like Receptor-1 is regulated in human myocardial infarction but its absence does not affect infarct size in mice**

Guilielmus H.J.M. Ellenbroek^1^, MD^^; Judith J. de Haan^1^, MSc^^; Bas R. van Klarenbosch^3^, MD; Maike A.D. Brans^1^, Sander M. van de Weg^1^; Mirjam B. Smeets^1^, PhD; Sanne de Jong^2^, PhD; Fatih Arslan^1,3^, MD, PhD; Leo Timmers^3^, MD, PhD; Marie-José T.H. Goumans^6^, PhD; Imo E. Hoefer^1,4^, MD, PhD; Pieter A. Doevendans^3,5^,, MD, PhD; Gerard Pasterkamp^1,4^, MD, PhD; Linde Meyaard^7^, PhD; Saskia C.A. de Jager^1,7^, PhD*

**Supplementary figure legends**

**Supplementary Fig. S1**

Gating strategy of flow cytometry on human blood samples from healthy controls and patients 3 days and 6 weeks after MI. After selection of the cell population based on FS/SS scatter, we selected leukocytes defined as CD45^+^. From this population, granulocytes were selected based on FS/SS scatter. Monocytes were selected based on a Boolean gate (termed Myeloid= ((Leukocytes AND (NOT granulocytes)), and subdivided into classical monocytes (CD14^+^CD16^-^), intermediate monocytes (CD14^+^/CD16^+^) and non-classical monocytes (CD14^-^/CD16^+^). The three monocyte subsets comprise the total monocyte population. LAIR-1 expression by MFI was determined on all leukocytes. *MI: myocardial infarction.*

**Supplementary Fig. S2**

Gating strategy of flow cytometry on mouse heart tissue. First CD45^+^ cells were selected and then, based on FS/SS scatter, the cell population was selected. To further improve purity of only CD45^+^ cells, an additional gating on CD45^+^/SSC was performed and cells were termed CD45^+^. Neutrophils were defined as CD11b^+^ and Ly6G^+^ within the CD45^+^ population. A Boolean gate (termed Not neutro = CD45^+^ AND (NOT Neutrophils)) was generated to exclude neutrophils from further analysis. Macrophages were defined as CD11b^+^ and F4/80 positive cells within the ‘Not neutro’ gate. Monocytes were selected based on a Boolean gate (input gate Mono = "Not neutro" AND (NOT Macrophages)), where Ly6C low and Ly6C high monocytes were defined as CD11b^+^ and LyC6^+^ or LyC6^++^ within the monocyte gate. T-cells were analysed in the ‘Not neutro’ population and defined as CD4^+^ and CD8^+^ T-cells.

**Supplementary Fig. S3**

Flow cytometry showed that LAIR-1 receptor expression on CD8^+^ T-lymphocytes was not different 3 days and 6 weeks after MI or compared to healthy controls. (22 patients and 20 healthy controls)

**Supplementary Fig. S4**

TNFα levels in the plasma does not increase after MI. There is no difference in TNFα levels in the plasma between WT and LAIR-1^-/-^ mice. IL-6 levels in the plasma increases 3 days after MI and decreases again 28 days after MI. There is no difference in IL-6 levels in the plasma between WT and LAIR-1^-/-^ mice. (0 days; N=2 WT and 2 LAIR-1^-/-^, 3 days; N=7 WT and 8 LAIR-1^-/-^, 28 days; N=11 WT and 10 LAIR-1^-/-^ per group) ** p<0.05, ** p<0.01, *** p<0.001*


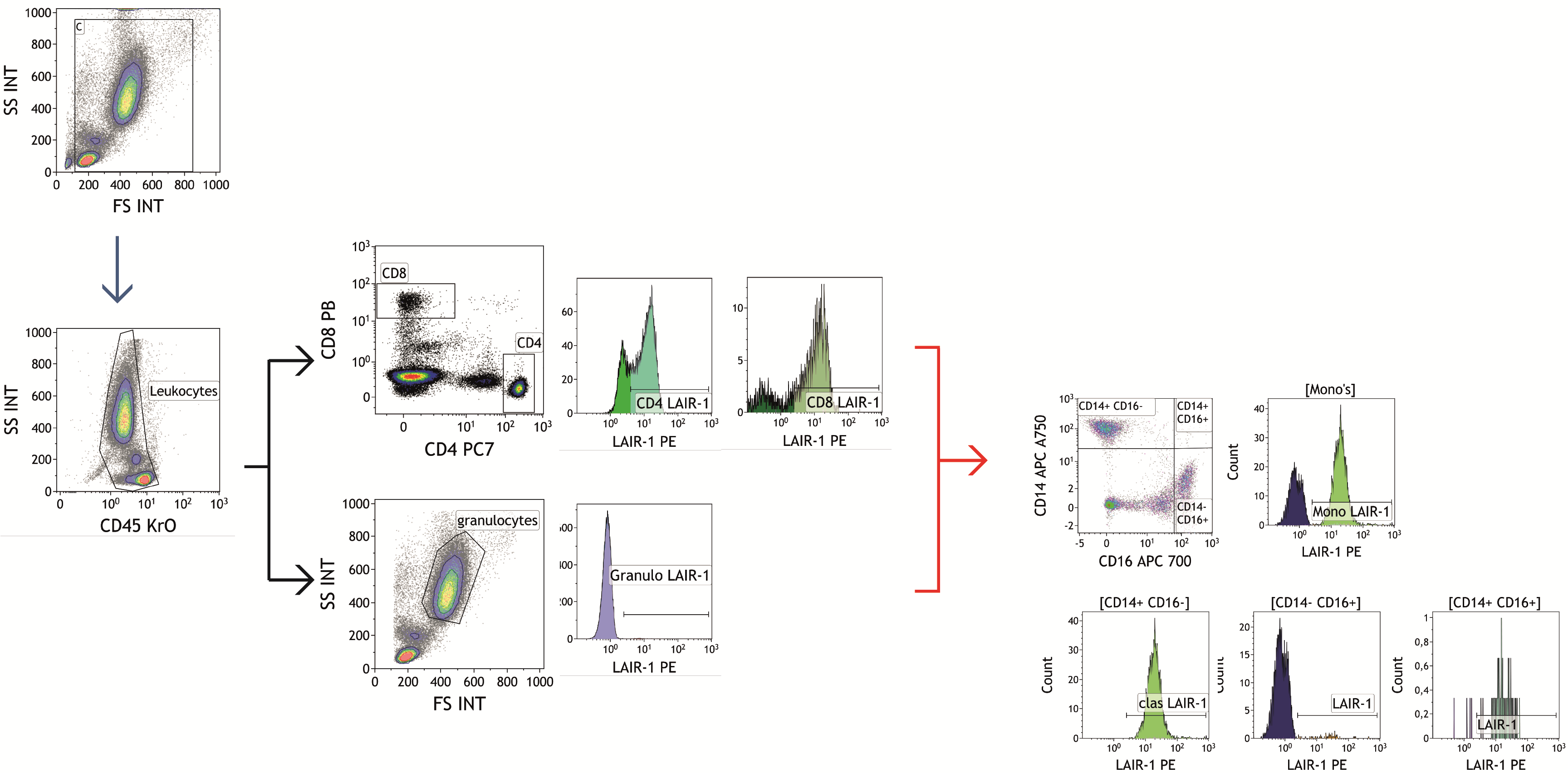
**Supplementary Fig. S1**

**Supplementary Fig. S2**

**
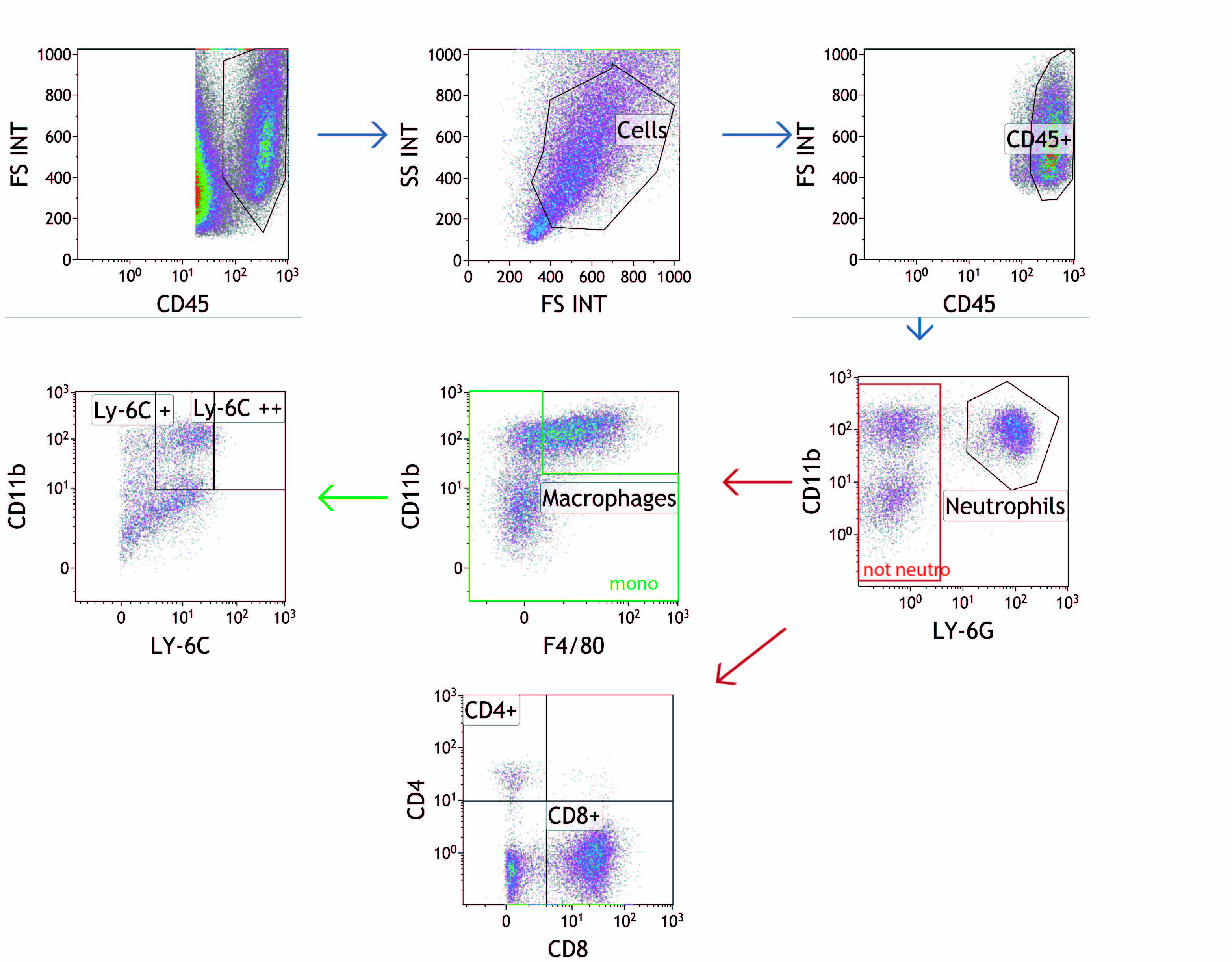
**

**Supplementary Fig. S3**

**Supplementary Fig. S4**

**
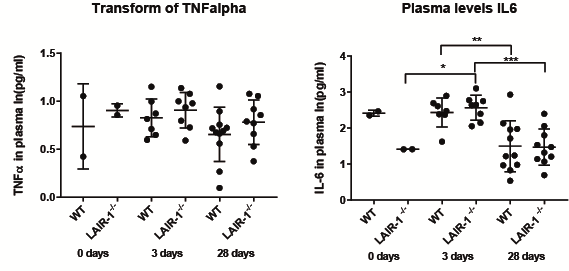
**

**Supplementary table legends**

**Supplementary Table S1**

Antibody mixture for human blood samples for flow cytometry on DEFI-MI patients and healthy volunteers.

**Supplementary Table S2**

Antibody mixture for murine blood, spleen and lymph node for flow cytometry on WT and LAIR-1^-/-^ mice at baseline, 3 days and 28 days after MI.

**Supplementary Table S3**

Antibody mixture for murine myocardial cells for flow cytometry on WT and LAIR-1^-/-^ mouse hearts at baseline and 3 days after MI.

Supplementary Table S4

MFI of LAIR-1 on different leukocytes in the blood at baseline, 3 days and 28 days after MI in WT mice. * p<0.05 compared to baseline

**Supplementary Table S5**

Leukocyte levels in the heart at baseline and after 3 days of MI in WT and LAIR-1^-/-^ mice. Percentages are given of total CD45^+^ cells.

**Supplementary Table S6**

Leukocyte levels in the blood at baseline, 3 days and 28 days after MI in WT and LAIR-1^-/-^ mice. Percentages are given of total leukocytes.

**Supplementary Table S7**

Leukocyte levels in the spleen at baseline, 3 days and 28 days after MI in WT and LAIR-1^-/-^ mice. Percentages are given of total leukocytes.

**Supplementary Table S8**

Leukocyte levels in the lymph nodes at baseline, 3 days and 28 days after MI in WT and LAIR-1^-/-^ mice. Percentages are given of total leukocytes.

**Supplementary Table S1:** Antibody mixture for human blood

| **Antibody** | **Flurophore** | **Clone** | **Company** | **µL/sample** |
| --- | --- | --- | --- | --- |
| LAIR | PE | DX26 | BD Pharmingen | 5 |
| CD16 | APC-A700 | 3G8 | Beckman Coulter | 2 |
| CD14 | APC-A750 | RMO52 | Beckman Coulter | 2 |
| CD45 | Krome Orange | J.33 | Beckman Coulter | 2 |

**Supplementary Table S2:** Antibody mixture for murine blood, spleen and lymph node

| **Antibody** | **Flurophore** | **Clone** | **Company** | **Concentration** | **µL/sample** |
| --- | --- | --- | --- | --- | --- |
| CD11b | A488 | M1/70 | eBioscience | 0.5 mg/ml | 0.125 |
| LAIR-1 | PE | 113 | eBioscience | 0.2 mg/ml | 0.3 |
| CD3 | PE-CF594 | 145-2C11 | BD Bioscience | 0.2 mg/ml | 0.5 |
| F4/80 | PE-CY7 | BM8 | eBioscience | 0.2 mg/ml | 0.2 |
| LY-6G | APC | 1A8 | eBioscience | 0.2 mg/ml | 0.125 |
| CD4 | Alexa Fluor 700 | GK1.5 | eBioscience | 0.2 mg/ml | 0.125 |
| CD8 | APC-eFluor 780 | 53-6.7 | eBioscience | 0.2 mg/ml | 0.125 |
| LY-6C | e450 | HK1.4 | eBioscience | 0.2 mg/ml | 0.125 |
| CD62L | Briljant Violet510 | MEL-14 | Biolegend | 0.05 mg/ml | 0.25 |

**Supplementary Table S3:** Antibody mixture for murine myocardial cells

| **Antibody** | **Flurophore** | **Clone** | **Company** | **Concentration** | **µL/sample** |
| --- | --- | --- | --- | --- | --- |
| CD11b | A488 | M1/70 | eBioscience | 0.5 mg/ml | 0.125 |
| CD62L | PE | MEL-14 | eBioscience | 0.2 mg/ml | 0.25 |
| CD45 | PE-CF594 | 30-F11 | BD Bioscience | 0.2 mg/ml | 0.5 |
| F4/80 | PE-CY7 | BM8 | eBioscience | 0.2 mg/ml | 0.2 |
| LY-6G | APC | 1A8 | eBioscience | 0.2 mg/ml | 0.125 |
| CD4 | Alexa Fluor 700 | GK1.5 | eBioscience | 0.2 mg/ml | 0.125 |
| CD8 | APC-eFluor 780 | 53-6.7 | eBioscience | 0.2 mg/ml | 0.125 |
| LY-6C | e450 | HK1.4 | eBioscience | 0.2 mg/ml | 0.125 |
| Sytox Blue |  |  | Life Technology | 1 mM solution | 0.1 |

Supplementary Table S4: MFI of LAIR-1 on different leukocytes

|  | **Baseline** | **3 days** | **28 days** |
| --- | --- | --- | --- |
| **Neutrophils** | 41.9 ± 9.4 MFI | 46.8 ± 5.5 MFI | 21.5 ± 3.4 MFI* |
| **Macrophages** | 64.0 ± 2.8 MFI | 43.1 ± 3.9 MFI* | 61.1 ± 13.2 MFI |
| **Ly6C High monocytes** | 24.4 ± 2.2 MFI | 24.1 ± 1.5 MFI | 25.9 ± 2.4 MFI |
| **Ly6C Low monocytes** | 54.0 ± 11.6 MFI | 38.1 ± 4.5 MFI | 44.1 ± 3.4 MFI |
| **CD4 T-cells** | 6.7 ± 0.2 MFI | 6.4 ± 0.3 MFI | 6.4 ± 0.4 MFI |
| **CD8 T-cells** | 9.1 ± 0.9 MFI | 8.7 ± 0.4 MFI | 10.4 ± 2.2 MFI |

**Supplementary Table S5**

| **Myocardium** | **Baseline** | | | **Remote Area** | | | **Infarct Area** | | |  |
| --- | --- | --- | --- | --- | --- | --- | --- | --- | --- | --- |
|  | | WT | LAIR-1 ^-/-^ | | WT | LAIR-1 ^-/-^ | | WT | LAIR-1 ^-/-^ | |
| **Neutrophils** | | 7.4±5.9 % | 2.5±1.8 % | | 38.7±16.6 % | 38.5±8.6 % | | 56.8±11.9 % | 49.4±14.3 % | |
| **Macrophages** | | 28.2±4.2 % | 23.1±3.2 % | | 38.0±14.0 % | 41.5±12.6 % | | 25.9±8.2 % | 30.9±8.7 % | |
| **Ly6C High monocytes** | | 0.3±0.1 % | 0.2±0.1 % | | 1.9±2.3 % | 2.6±3.9 % | | 3.0±1.9 % | 4.5±1.8 % | |
| **Ly6C Low monocytes** | | 1.9±0.4 % | 2.6±1.0 % | | 5.6±2.9 % | 5.8±2.7 % | | 3.9±2.4 % | 4.6±2.9 % | |
| **CD4 T-cells** | | 8.0±1.1 % | 7.7±0.6 % | | 3.5±2.4 % | 4.3±3.2 % | | 1.9±1.1 % | 2.0±1.2 % | |
| **CD8 T-cells** | | 6.3±0.5 % | 5.9±0.9 % | | 47.1±19.0 % | 51.7±12.3 % | | 34.3±6.8 % | 42.5±10 % | |

| **Blood** | **Baseline** | | | **3 days** | | | **28 days** | | |  |
| --- | --- | --- | --- | --- | --- | --- | --- | --- | --- | --- |
|  | | WT | LAIR-1 ^-/-^ | | WT | LAIR-1 ^-/-^ | | WT | LAIR-1 ^-/-^ | |
| **Neutrophils** | | 24.5±12.3 % | 10.5±6.9 % | | 28.1±23.5 % | 22.6±8.3 % | | 4.9±1.2 % | 20.6±17.0 % | |
| **Macrophages** | | 7.2±3.3 % | 7.0±2.5 % | | 5.4±4.4 % | 7.1±4.0 % | | 3.7±0.9 % | 5.8±1.9 % | |
| **Ly6C High monocytes** | | 5.0±3.2 % | 3.5±1.5 % | | 2.0±1.5 % | 3.0±2.0 % | | 1.1±0.3 % | 2.7±2.3 % | |
| **Ly6C Low monocytes** | | 2.2±0.3 % | 3.0±0.6 % | | 4.5±0.6 % | 5.0±1.2 % | | 3.5±0.3 % | 3.9±0.9 % | |
| **CD4 T-cells** | | 13.1±3.3 % | 14.8±1.6 % | | 9.0± 2.6 % | 11.4±3.2 % | | 8.4±2.5 % | 9.8±1.6 % | |
| **CD8 T-cells** | | 9.4±2.2 % | 10.7±1.5 % | | 8.7±3.8 % | 10.9±2.5 % | | 8.8±3.0 % | 10.0±2.4 % | |

**Supplementary Table S6**

**Supplementary Table S7**

| **Spleen** | **Baseline** | | | **3 days** | | | **28 days** | | |  |
| --- | --- | --- | --- | --- | --- | --- | --- | --- | --- | --- |
|  | | WT | LAIR-1 ^-/-^ | | WT | LAIR-1 ^-/-^ | | WT | LAIR-1 ^-/-^ | |
| **CD4 T-cells** | | 17.3±2.7 % | 15.8±1.0 % | | 13.9±2.8 % | 11.3±2.1 % | | 17.3±2.4 % | 16.3±2.0 % | |
| ***Tcm*** | | 72.6±3.2 % | 73.8±1.5 % | | 66.2±8.8 % | 71.8±6.7 % | | 66.6±5.7 % | 77.6±2.4 % | |
| ***Tem*** | | 27.5±3.2 % | 26.2±1.5 % | | 33.8±8.7 % | 28.1±6.7 % | | 33.3±5.7 % | 22.3±2.4 % | |
| **CD8 T-cells** | | 11.9±1.4 % | 11.6±0.8 % | | 9.8±2.5 % | 9.2±2.2 % | | 12.5±1.4 % | 12.4±0.8 % | |
| ***Tcm*** | | 94.8±0.7 % | 95.0±0.8 % | | 94.1±1.4 % | 93.9±1.9 % | | 93.9±2.8 % | 96.0±0.6 % | |
| ***Tem*** | | 3.6±1.1 % | 2.8±0.5 % | | 3.7±1.1 % | 5.0±1.8 % | | 3.1±0.9 % | 3.4±1.3 % | |

**Supplementary Table 8**

| **Lymph node** | **Baseline** | | | **3 days** | | | **28 days** | | |  |
| --- | --- | --- | --- | --- | --- | --- | --- | --- | --- | --- |
|  | | WT | LAIR-1 ^-/-^ | | WT | LAIR-1 ^-/-^ | | WT | LAIR-1 ^-/-^ | |
| **CD4 T-cells** | | 21.1±6.2 % | 17.8±4.9 % | | 11.4±2.8 % | 11.3±2.8 % | | 16.1±4.5 % | 15.3±2.9 % | |
| ***Tcm*** | | 86.6±2.6 % | 86.4±3.0 % | | 78.5±5.4 % | 82.6±4.2 % | | 65.4±7.1 % | 76.8±5.4 % | |
| ***Tem*** | | 13.5±2.7 % | 13.6±3.0 % | | 21.5±5.3 % | 17.2±4.3 % | | 34.7±7.1 % | 23.2±5.4 % | |
| **CD8 T-cells** | | 16.5±4.6 % | 14.9±5.1 % | | 11.2±2.8 % | 12.2±3.4 % | | 13.9±3.6 % | 14.6±2.5 % | |
| ***Tcm*** | | 97.8±0.8 % | 97.9±0.8 % | | 96.8±0.8 % | 97.0±1.4 % | | 97.5±0.6 % | 97.7±0.6 % | |
| ***Tem*** | | 0.8±0.1 % | 0.7±0.2 % | | 2.0±0.9 % | 2.4±1.2 % | | 0.9±0.3 % | 1.3±0.2 % | |
